# Integrated inflammatory-immune-nutritional signatures differentiate lung phenotypes in systemic sclerosis

**DOI:** 10.3389/fmed.2026.1795461

**Published:** 2026-04-14

**Authors:** Huichen Luo, Danhui Hu, Xu Wang, Daming Ou, Ling Lei

**Affiliations:** 1 Department of Rheumatology and Immunology, The First Affiliated Hospital of Guangxi Medical University, Nanning, Guangxi, China; 2 Department of Rheumatology and Immunology, The First Affiliated Hospital of University of South China, Hengyang, Hunan, China; 3 Department of Neonatology, The First Affiliated Hospital of University of South China, Hengyang, Hunan, China

**Keywords:** biomarkers, interstitial lung disease, phenotyping, pulmonary arterial hypertension, systemic sclerosis

## Abstract

**Background:**

Systemic sclerosis (SSc) exhibits heterogeneous pulmonary involvement, most commonly interstitial lung disease (ILD) and pulmonary arterial hypertension (PAH). How systemic inflammatory, immune, and nutritional states differ across these lung phenotypes remains incompletely understood.

**Objective:**

To characterize integrated inflammatory-immune-nutritional signatures across SSc lung phenotypes and validate their robustness using multivariable modeling and unsupervised clustering.

**Methods:**

We conducted a retrospective cross-sectional study of 314 consecutive SSc inpatients fulfilling the 2013 ACR/EULAR criteria (2018–2023). Patients were stratified into four lung phenotypes: ILD-/PAH- (*n* = 45), ILD + /PAH- (*n* = 183), ILD-/PAH + (*n* = 12), and ILD + /PAH + (n = 74). Routine laboratory data were used to derive inflammatory markers (hs-CRP, ESR, NLR, PLR, MLR, SII, SIRI, SCI), nutritional parameters (BMI, albumin, lipid profiles), and immune markers (autoantibodies, immunoglobulins, complement). We performed (1) group comparisons; (2) multinomial logistic regression focusing on the three predominant phenotypes (ILD-/PAH- as reference; ILD + /PAH-; ILD + /PAH +), excluding ILD-/PAH + due to small sample size (*n* = 12) and sparse-data instability; and (3) k-means clustering of standardized biomarker profiles. Internal validation and clustering stability analyses were performed.

**Results:**

ILD + /PAH + patients showed a higher systemic inflammatory burden, including higher ESR (38 [21–61.5] vs. 23 [10–45] mm/h, *p* = 0.008), hs-CRP (10.15 [3.34–28.18] vs. 5.9 [1.5–16.1] mg/L, *p* = 0.017), CRP/albumin ratio (0.33 [0.11–0.95] vs. 0.17 [0.04–0.49], *p* = 0.019), and SII (1124.76 [700.8–2034.14] vs. 820.34 [461.33–1197.08], *p* = 0.013) compared with ILD-/PAH-. Anti-Scl-70 positivity was enriched in ILD-containing phenotypes (*p* < 0.001), and immunoglobulins varied (IgG *p* = 0.032; IgA *p* = 0.028). In multinomial models, female sex and longer disease duration were associated with both ILD + /PAH- (OR 3.48, 95%CI 1.51–8.04; OR 1.02 per month, 95%CI 1.01–1.04) and ILD + /PAH + (OR 3.49, 95%CI 1.31–9.33; OR 1.03 per month, 95%CI 1.01–1.05). Triglycerides (Z-score) were associated with ILD + /PAH- (OR 1.70, 95%CI 1.05–2.77), while log(ESR) (Z-score) was associated with ILD + /PAH + (OR 1.82, 95%CI 1.01–3.27). Clustering supported a three-cluster structure driven by inflammation (ESR), mucosal immunity (IgA), and metabolic dysregulation (triglycerides), with strong stability (Adjusted Rand Index = 1.00). Internal validation showed stable within-cohort performance (accuracy ∼0.62–0.65).

**Conclusion:**

SSc lung phenotypes defined by ILD/PAH co-occurrence display distinct integrated inflammatory-immune-nutritional signatures. The ILD + /PAH + phenotype reflects a high-burden systemic inflammatory state, and a small set of coherent markers (ESR, triglycerides, IgA, and autoantibodies) provides a reproducible framework for phenotype-oriented stratification.

## Graphical Abstract

**Figure:**
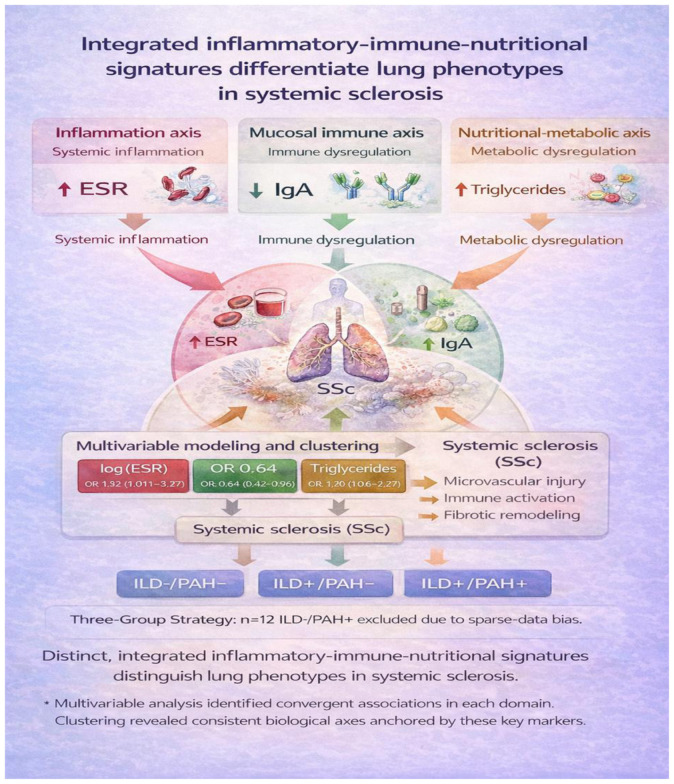
Overview of the study design and integrated inflammatory–immune–nutritional signatures differentiating SSc lung phenotypes defined by ILD/PAH co-occurrence. SSc, systemic sclerosis; ILD, interstitial lung disease; PAH, pulmonary arterial hypertension; ESR, erythrocyte sedimentation rate; SII, systemic immune-inflammation index.

## Highlights

1. We defined four clinically relevant lung phenotypes in SSc based on the co-occurrence patterns of interstitial lung disease (ILD) and pulmonary arterial hypertension (PAH), and systematically characterized their integrated inflammatory-immune-nutritional signatures.
2. A prespecified analytical hierarchy combining descriptive comparisons, multinomial logistic regression, and exploratory clustering was applied to delineate phenotype-specific biomarker profiles.
3. Patients with combined ILD and PAH (ILD + /PAH +) demonstrated the highest systemic inflammatory burden and distinct immune–nutritional features, supporting their classification as a high-burden systemic phenotype rather than a simple additive comorbidity.
4. Multinomial regression and unsupervised clustering converged on a small, biologically coherent set of signals (e.g., ESR, triglycerides, IgA, and autoantibodies), underscoring their value for phenotypic stratification rather than individual-level prediction.

## Introduction

Systemic sclerosis (SSc) is a complex autoimmune connective tissue disease characterized by microvascular injury, immune dysregulation, and progressive fibrosis affecting multiple organs (1, 2). Pulmonary involvement, particularly interstitial lung disease (ILD) and pulmonary arterial hypertension (PAH), is the leading cause of SSc-related morbidity and mortality (3–5). Current clinical assessment of SSc lung involvement relies heavily on imaging, hemodynamic evaluation, and pulmonary function testing. However, these tools capture structural and functional consequences rather than upstream systemic processes that might distinguish biologically meaningful lung phenotypes.

In routine practice and most observational studies, ILD and PAH have been investigated as separate complications or dichotomous outcomes (present/absent). In reality, patients may present with isolated ILD, isolated PAH, combined ILD and PAH, or neither complication, yielding four distinct patterns of pulmonary involvement (6, 7). Existing biomarker studies in SSc have predominantly focused on single inflammatory indices (such as CRP, NLR or ESR) or individual autoantibodies, often without considering how these markers behave across different ILD/PAH combinations. Furthermore, nutritional and metabolic indicators—including body mass index (BMI), albumin, and lipid profiles—are increasingly recognized as integrative markers of chronic inflammation, catabolic state, and cardiovascular risk (8–12), yet their role within SSc lung phenotypes has been largely overlooked. Thus, the interplay between inflammatory, immune, and nutritional domains across clinically defined lung phenotypes remains poorly characterized. The autoimmune component of SSc is marked by distinct autoantibody profiles that correlate with organ involvement. Anti-Scl-70 (topoisomerase I) is strongly associated with diffuse cutaneous SSc and ILD risk, whereas anti-centromere antibody (ACA) is more closely linked to limited cutaneous disease and PAH development (13–15). However, it is not known whether these autoantibody patterns, when considered together with systemic inflammatory and nutritional markers, form reproducible “signatures” that differentiate ILD-/ PAH-, ILD + / PAH-, ILD-/PAH + , and ILD + /PAH + phenotypes. Clarifying these signatures could help bridge the gap between pathobiology and clinically observable lung patterns. Previous SSc studies have rarely examined ILD and PAH jointly across all four combinations, and integrated inflammatory, immune, and nutritional domains into reproducible multi-marker signatures for lung phenotyping.

In this study, we conceptualized an integrated inflammatory-immune-nutritional signature framework to characterize SSc lung phenotypes. We hypothesized that the four ILD/PAH phenotypes would display quantifiably distinct systemic signatures, reflecting different degrees and patterns of immune activation, inflammation-related catabolism, and nutritional-metabolic compromise. Rather than developing a predictive tool, our primary objective was phenotypic classification and mechanistic characterization: (1) to compare biomarker distributions across the four lung phenotypes; (2) to identify biomarkers independently associated with each phenotype using multinomial logistic regression; and (3) to evaluate whether data-driven clustering of biomarker profiles recapitulates or extends clinically defined lung phenotypes.

## Materials and methods

### Study design and population

We conducted a retrospective cross-sectional study of SSc inpatients at The First Affiliated Hospital of Guangxi Medical University between December 2018 and December 2023. The study was approved by the institutional Ethics Committee (No.2026-E0049) and conducted in accordance with the Declaration of Helsinki.

**FIGURE 1:**
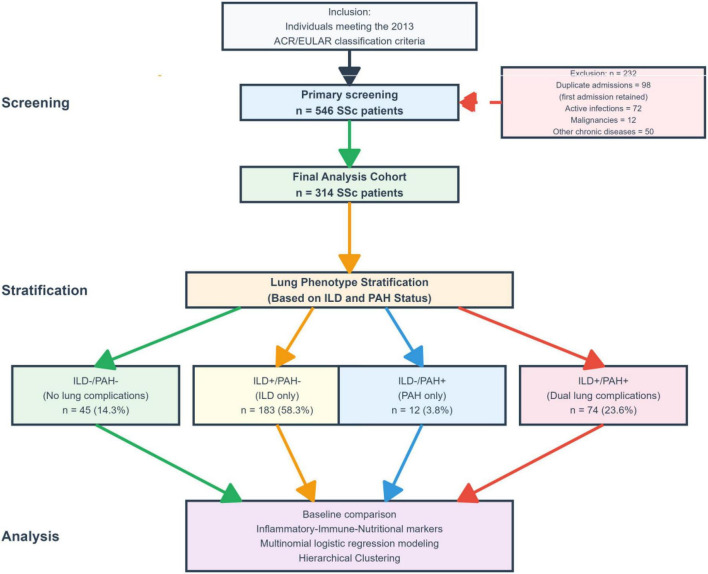
Flowchart of study design and SSc-ILD/PAH stratification. *Other major non–SSc chronic comorbidities likely to affect inflammatory/coagulation markers (e.g., advanced chronic kidney disease, chronic liver disease/cirrhosis, chronic pulmonary disease, or chronic heart failure).

From 546 screened patients, we included individuals who: (1) fulfilled the 2013 ACR/EULAR classification criteria for SSc (16); (2) underwent comprehensive pulmonary assessment allowing evaluation of both ILD and PAH; and (3) had complete laboratory data for key inflammatory, nutritional, and immune markers within 24 h of admission. We excluded: (1) duplicate admissions (retaining the first admission only); (2) active infection at admission; (3) concurrent malignancies; and (4) other chronic diseases that could substantially affect inflammatory markers. The final analysis cohort comprised 314 patients (Figure 1).

### Lung phenotype stratification

Patients were stratified into four mutually exclusive lung phenotypes based on ILD and PAH status:

ILD-/PAH- (*n* = 45, 14.3%): no lung complications, ILD + /PAH- (*n* = 183, 58.3%): ILD only, ILD-/PAH + (*n* = 12, 3.8%): PAH only, ILD + /PAH + (*n* = 74, 23.6%): combined ILD and PAH. ILD was diagnosed using high-resolution computed tomography (HRCT) by experienced thoracic radiologists, based on patterns consistent with SSc-Sc was diagnosed using high-resolution computed tomography (HRCT) by experienced thoracic radiologists, based(> 40 mmHg) and/or right heart catheterization where available, in line with contemporary guidelines (17, 18). This approach reflects real-world practice in our center, where echocardiography serves as the primary screening tool, and captures patients with clinical pulmonary hypertension regardless of hemodynamic classification (PAH and PAH-ILD).

### Clinical data collection

Clinical data were extracted from electronic medical records and included demographics, disease duration, SSc subtype (diffuse vs. limited), modified Rodnan skin score (mRSS), and organ involvement (digital ulcers, renal crisis, musculoskeletal and gastrointestinal involvement, and cardiovascular manifestations). Disease duration was calculated from the first non-Raynaud symptom to the date of index admission. Treatment history was recorded but not included in multivariable models due to heterogeneity and missingness.

### Laboratory measurements

Fasting venous blood samples were obtained within 24 h of admission. Inflammatory markers included: high-sensitivity C-reactive protein (hs-CRP), erythrocyte sedimentation rate (ESR), complete blood count with differential, and derived indices: neutrophil-to-lymphocyte ratio (NLR), platelet-to-lymphocyte ratio (PLR), monocyte-to-lymphocyte ratio (MLR), systemic immune-inflammation index (SII; platelet count × neutrophil count/lymphocyte count), systemic inflammation response index (SIRI; neutrophil count × monocyte count/lymphocyte count), and systemic inflammation index (SCI; composite index derived from leukocyte and lymphocyte counts). Nutritional and metabolic markers included: BMI, serum albumin, total cholesterol, triglycerides, high-density lipoprotein (HDL) cholesterol, and low-density lipoprotein (LDL) cholesterol. Immune markers included: antinuclear antibody (ANA), anti-Scl-70, ACA, anti-Ro-52, immunoglobulins (IgG, IgA, IgM), and complement components C3 and C4. All measurements were performed in the hospital’s central laboratory using standardized protocols and quality control procedures.

### Statistical analysis

Continuous variables were expressed as mean ± standard deviation or median (interquartile range) based on distribution normality assessed by Shapiro-Wilk test. Categorical variables were presented as frequencies and percentages. Group comparisons were performed using ANOVA or Kruskal-Wallis test for continuous variables and χ^2^ or Fisher’s exact test for categorical variables.

Our analytical strategy followed a rigorous “description-association-validation” hierarchy, which was explicitly phenotype-oriented rather than prediction-oriented. First, we used descriptive and univariate analyses to compare clinical characteristics and biomarker distributions across lung phenotypes, aiming to delineate phenotype-specific patterns. Second, we constructed nested multinomial logistic regression models to identify independent predictors. To ensure statistical robustness and avoid sparse-data bias, the small ILD-/PAH + subgroup (*n* = 12) was excluded from the multivariable modeling, focusing the analysis on the three predominant phenotypes (ILD-/ PAH-, ILD + / PAH-, ILD + /PAH +). Variables were entered progressively: Model 1 (Clinical Baseline), Model 2 (+ Nutritional), Model 3 (+ Inflammatory), and Model 4 (+ Immune). Continuous variables were standardized (Z-scores) or log-transformed (log + Z-score) to facilitate direct comparison of odds ratios (ORs). Third, internal validation of the final regression model was performed using bootstrap resampling (*n* = 100) and 5-fold cross-validation. Finally, unsupervised k-means clustering (*k* = 3) was applied to the standardized biomarker dataset to evaluate whether data-driven biological partitions aligned with clinical lung phenotypes. Cluster stability was verified using the Gap statistic and Jaccard bootstrap analysis. Internal validation of multinomial models focused on classification performance within this cohort rather than external prediction. All analyses were performed using R software (version 4.4.1) with significance set at *p* < 0.05.

## Results

### Baseline and systemic burden

The final cohort included 314 SSc patients with a mean age of 54.9 ± 10.7 years; 65.3% were female. Lung phenotypes were distributed as follows: ILD-/PAH- (*n* = 45, 14.3%), ILD + /PAH- (*n* = 183, 58.3%), ILD-/PAH + (*n* = 12, 3.8%), and ILD + /PAH + (*n* = 74, 23.6%) (Figure 1). Baseline characteristics differed across phenotypes (Table 1). Female sex varied substantially (*p* < 0.001), with the lowest proportion in ILD-/PAH + (16.7%) and higher proportions in ILD + /PAH- (70.5%) and ILD + /PAH + (71.6%). Disease duration also differed (*p* = 0.003), with the ILD + /PAH + group having the longest duration [median 24 (9.5–81) months]. Skin involvement (mRSS) differed across phenotypes (*p* = 0.003), with higher mRSS in ILD + /PAH + [median 15 (6.2–23)] compared with ILD-/PAH- [8 (4–12)] and ILD + /PAH- [10 (6–16)]. Gastrointestinal involvement was more frequent in ILD + /PAH + (32.4%) (*p* = 0.007). BMI differed across groups (*p* = 0.012), with the lowest mean BMI observed in ILD-/PAH + (19.4 ± 2.7 kg/m^2^). Clinically defined ILD/PAH co-occurrence phenotypes reflect a gradient of systemic burden, with ILD + /PAH + characterized by longer disease duration, higher skin involvement, and greater gastrointestinal involvement.

**TABLE 1:** Baseline clinical characteristics by lung phenotype.

Variable N	Overall 314	ILD-/PAH- 45	ILD + /PAH- 183	ILD-/PAH + 12	ILD + /PAH + 74	*P*- value
Age, years	54.9 ± 10.7	52.9 ± 12.2	54.5 ± 10.1	59.8 ± 11.4	56 ± 10.7	0.180
Female, n (%)	205 (65.3)	21 (46.7)	129 (70.5)	2 (16.7)	53 (71.6)	**< 0.001**
Zhuang ethnicity, n (%)	177 (56.4)	28 (62.2)	98 (53.6)	7 (58.3)	44 (59.5)	0.677
Disease duration, months	13 (6–48)	12 (4–36)	12 (6–36)	8.5 (3–31.2)	24 (9.5–81)	**0.003**
Smoking, n (%)	77 (24.5)	15 (33.3)	41 (22.4)	6 (50)	15 (20.3)	0.064
Drinking, n (%)	69 (22)	16 (35.6)	34 (18.6)	4 (33.3)	15 (20.3)	0.069
BMI, kg/m^2^	20.9 ± 3.3	21.6 ± 4.8	21.2 ± 3.1	19.4 ± 2.7	20 ± 2.6	**0.012**
Diffuse SSc, n (%)	213 (67.8)	25 (55.6)	126 (68.9)	6 (50)	56 (75.7)	0.071
MRSS score	10 (6–18)	8 (4–12)	10 (6–16)	12 (5.5–19)	15 (6.2–23)	**0.003**
Digital ulcer, n (%)	80 (25.5)	11 (24.4)	44 (24)	1 (8.3)	24 (32.4)	0.265
Necrosis/gangrene, n (%)	47 (15)	8 (17.8)	27 (14.8)	1 (8.3)	11 (14.9)	0.873
Renal crisis, n (%)	9(2.9)	0 (0)	8 (4.4)	1 (8.3)	0 (0)	0.098
Joint impairment, n (%)	131 (41.7)	16 (35.6)	75 (41)	5 (41.7)	35 (47.3)	0.639
Muscle damage, n (%)	71 (22.6)	8 (17.8,	39 (21.4)	4 (33.3)	20 (27)	0.502
Gastrointestinal involvement, n (%)	59 (18.8)	7 (15.6)	27 (14.8)	1 (8.3)	24 (32.4)	**0.007**
Cardiovascular involvement, n (%)	304 (96.8)	41 (93.2)	180 (98.4)	12 (100)	71 (95.9)	0.243

Data are presented as mean ± SD, median (IQR), or n (%). *P*-values represent overall group comparisons using ANOVA/Kruskal-Wallis test for continuous variables and x^2^/Fisher’s exact test for categorical variables. ILD, interstitial lung disease; PAH, pulmonary arterial hypertension; SSc, systemic sclerosis; BMI, body mass index; MRSS, modified Rodnan skin score. Bold values indicate statistically significant differences (*P* < 0.05).

### Inflammatory and nutritional signatures: the “inflamed and catabolic” phenotype

Inflammatory and nutritional markers showed phenotype-specific differences (Table 2). The ILD + /PAH + phenotype demonstrated the highest inflammatory activity across multiple measures, including higher ESR [38 (21–61.5) vs. 23 (10–45) mm/h in ILD-/ PAH-, *p* = 0.008] and higher hs-CRP [10.15 (3.34–28.18) vs. 5.9 (1.5–16.1) mg/L, *p* = 0.017). The CRP/albumin ratio also differed and was highest in ILD + /PAH + [0.33 (0.11–0.95), *p* = 0.019]. Composite inflammatory indices further supported phenotype differences. SII differed across groups (*p* = 0.013) and was higher in ILD + /PAH + [1124.76 (700.8–2034.14)] than in ILD-/PAH- [820.34 (461.33–1197.08)]. In contrast, several cell-ratio indices showed weaker between-group separation at the univariate level (e.g., NLR *p* = 0.078; PLR *p* = 0.133; MLR *p* = 0.647). Nutritional–metabolic markers varied across phenotypes. Triglycerides differed (*p* = 0.028), as did LDL cholesterol (*p* = 0.042). Albumin showed a borderline overall difference (*p* = 0.051), with the lowest mean albumin in ILD + /PAH + (32.18 ± 4.72 g/L). HDL cholesterol did not differ significantly across phenotypes in Table 2 (*p* = 0.648). ILD + /PAH + is consistently associated with higher systemic inflammatory burden (ESR, hs-CRP, CRP/Alb, SII), while metabolic markers (notably triglycerides and LDL) provide additional, partially independent phenotype differentiation.

**TABLE 2:** Inflammatory and nutritional markers by lung phenotype.

Variable N	Overall 314	ILD-/PAH- 45	ILD + /PAH- 183	ILD-/PAH + 12	ILD + /PAH + 74	*P* value
Nutritional markers						
BMI, kg/m^2^	20.88 ± 3.34	21.61 ± 4.81	21.17 ± 3.13	19.44 ± 2.66	19.97 ± 2.57	**0.012**
Albumin, g/L	33.28 ± 4.89	34.43 ± 3.83	33.36 ± 5.1	34.53 ± 5.36	32.18 ± 4.72	0.051
Hemoglobin, g/L	113.55 ± 20.24	114.98 ± 22.01	114.76 ± 19.04	117.75 ± 25.15	108.94 ± 20.89	0.193
Total cholesterol,	4.34 (3.71–5.09)	4.16 (3.62–4.78)	4.46 (3.77–5.23)	4.14 (3.73–4.43)	4.34 (3.51–5.04)	0.052
mmol/L						
Triglycerides, mmol/L	1.42 (1.03–1.93)	1.43 (1.1–1.67)	1.55 (1.12–2.03)	1.16 (1.09–2.05)	1.24 (0.94–1.74)	**0.028**
HDL cholesterol,	1.01 (0.87–1.21)	1.04 (0.84–1.22)	1.02 (0.88–1.22)	0.95 (0.9–1.14)	0.99 (0.81–1.15)	0.648
mmol/L						
LDL cholesterol, mmol/L	2.53 (2.06–3.14)	2.4 (1.98–2.9)	2.69 (2.15–3.28)	2.22 (1.83–2.7)	2.47 (2.03–3.11)	**0.042**
Basic inflammatory markers						
ESR, mm/h	28 (16–48.75)	23 (10–45)	26 (14–44)	26.5 (14.5–42.5)	38 (21–61.5)	**0.008**
hs-CRP, mg/L	6.47 (2.32–18)	5.9 (1.5–16.1)	5.4 (2.13–14.79)	3.21 (2.55–13.34)	10.15 (3.34–28.18)	**0.017**
CRP/Albumin ratio	0.2 (0.07–0.57)	0.17 (0.04–0.49)	0.16 (0.07–0.48)	0.09 (0.07–0.51)	0.33 (0.11–0.95)	**0.019**
Cell Ratio inflammatory markers						
Neutrophil-to-	3.38 (2.31–5.21)	2.77 (2.06–3.87)	3.43 (2.35–5)	3.4 (2.41–6.48)	3.65 (2.55–6.29)	0.078
lymphocyte ratio						
Platelet-to-lymphocyte	203.63 (134.22-	180.25 (119.43-	205.4 (134.23-	178.57 (120.15-	207.4 (147.11–	0.133
Ratio	306.37)	253.26)	301.48)	337.5)	370)	
Monocyte-to-lymphocyte	0.48 (0.33–0.69)	0.42 (0.32–0.64)	0.49 (0.34–0.66)	0.36 (0.28–0.74)	0.49 (0.34–0.74)	0.647
Ratio						
Composite inflammatory indices						
Systemic immune-	956.52 (563.05-	820.34 (461.33-	954.94 (560.54-	772.75 (476.06-	1124.76 (700.8-	**0.013**
inflammation index	1636.99)	1197.08)	1581.77)	1899.71)	2034.14)	
Systemic inflammation	2.2 (1.34–3.71)	2.07 (1–3.02)	2.2 (1.37–3.69)	1.99 (1.19–3.87)	2.12 (1.47–4.57)	0.331
response index						
Systemic inflammation	147.59 (101.99-	124.22 (99.93-	143.4 (99.31-	119.68 (78.53-	165.44 (115.5-	0.094
Index	203.21)	212.4)	197.97)	155.49)	210.4)	

Data are presented as mean ± SD for normally distributed variables or median (IQR) for non-normally distributed variables. *P-*values represent overall group comparisons using Kruskal-Wallis test. ESR, erythrocyte sedimentation rate; hs-CRP, high-sensitivity C-reactive protein; HDL, high-density lipoprotein; LDL, low-density lipoprotein; NLR, neutrophil-to-lymphocyte ratio; PLR, platelet-to-lymphocyte ratio; MLR, monocyte-to-lymphocyte ratio; SII, systemic immune- inflammation index; SIRI, systemic inflammation response index; SCI, systemic inflammation index. Bold values indicate statistically significant differences (*P* < 0.05).

### Immune profiling: autoantibodies define the track

Immune profiles further differentiated lung phenotypes (Table 3). Anti-Scl-70 positivity differed markedly across groups (*p* < 0.001) and was enriched in ILD-containing phenotypes (ILD + /PAH- 79.8%; ILD + /PAH + 78.4%), compared with ILD-/PAH- (61.4%) and ILD-/PAH + (33.3%). ACA did not differ significantly by phenotype in the univariate comparison (*p* = 0.129), but its distribution pattern remained clinically informative when interpreted alongside regression modeling. Immunoglobulins also differed: IgG varied across groups (*p* = 0.032), and IgA differed (*p* = 0.028). IgM did not differ significantly (*p* = 0.358). Complement components C3 and C4 were not significantly different across phenotypes (C3 *p* = 0.308; C4 *p* = 0.639). ILD-containing phenotypes are strongly associated with anti-Scl-70 positivity, and humoral immune markers (IgG/IgA) add an additional layer of phenotype differentiation beyond autoantibodies alone.

**TABLE 3:** Immune markers by lung phenotype.

Variable N	Overall 314	ILD-/PAH- 45	ILD + /PAH- 183	ILD-/PAH + 12	ILD + /PAH + 74	*P*-value
Autoantibodies, n (%)						
ANA	304 (96.8)	41 (93.2)	180 (98.4)	12 (100)	71 (95.9)	0.208
Anti-Scl-70	235 (74.8)	27 (61.4)	146 (79.8)	4 (33.3)	58 (78.4)	**< 0.001**
Anti-centromere	27 (8.6)	7 (15.9)	12 (6.6)	2 (16.7)	6 (8.1)	0.129
Anti-Ro-52	79 (25.2)	10 (23.3)	41 (22.4)	4 (33.3)	24 (32.4)	0.322
Immunoglobulins						
IgG, g/L	15.57 (12.1819.25)	14.9 (12.21–18.74)	15.03 (11.62–19.03)	15.28 (12.09–21.63)	16.8 (13.35–22.18)	**0.032**
IgA, g/L	2.53 (1.87–3.38)	2.5 (2.08–3.45)	2.38 (1.77–3.23)	3.04 (2.59–3.5)	2.64 (2.19–3.56)	**0.028**
igM, g/L	1.25 (0.82–1.86)	1.28 (0.78–1.88)	1.21 (0.78–1.89)	0.86 (0.61–1.35)	1.27 (0.91–1.83)	0.358
**Complement**						
C3, g/L	1.13 ± 0.24	1.11 ± 0.24	1.13 ± 0.24	1.0 4 ± 0.21	1.14 ± 0.23	0.308
C4, g/L	0.31 ± 0.12	0.31 ± 0.13	0.31 ± 0.12	*^r^*.27. 0.1	0.3 ± 0.1	0.639

Data are presented as n (%) for categorical variables or median (IQR) for continuous variables. *P*-values represent overall group comparisons using X^2^/Fisher’s exact test for categorical variables and Kruskal-Wallis test for continuous variables. Bold values indicate statistically significant differences (*P* < 0.05).

### Multivariable multinomial logistic regression (optimized 3-group model)

To quantify independent associations with phenotype classification while minimizing sparse-data instability, multinomial regression focused on the three predominant phenotypes (ILD-/PAH- reference; ILD + /PAH-; ILD + /PAH +). The ILD-/PAH + subgroup (*n* = 12) was presented descriptively in Tables 1–3 but excluded from multivariable modeling due to limited sample size and risk of unstable estimates (sparse-data bias/separation), consistent with Table 4. After adjustment, several robust clinical correlates were shared across ILD-involving phenotypes. Female sex was associated with higher odds of both ILD + /PAH- (OR 3.48, 95%CI 1.51–8.04, *p* = 0.004) and ILD + /PAH + (OR 3.49, 95%CI 1.31–9.33, *p* = 0.013) relative to ILD-/PAH-. Longer disease duration was also associated with both ILD + /PAH- (OR 1.02 per month, 95%CI 1.01–1.04, *p* = 0.009) and ILD + /PAH + (OR 1.03 per month, 95%CI 1.01–1.05, *p* = 0.001).

**TABLE 4:** Multivariable multinomial logistic regression (optimized model).

Variable	ILD + /PAH- vs. ILD-/PAH- OR (95% CI)	*P-*value	ILD + /PAH + vs. ILD-/PAH- OR (95% CI)	*P-*value
Sample size	183 vs. 45		74 vs. 45	
Excluded group (minimal disclosure)	ILD-/PAH + (*n* = 12) excluded from modeling		Due to limited sample size and sparse-data bias risk	Shown descriptively in Tables 1–3
Baseline characteristics (original values)				
Age, per year	1.01 (0.97–1.05)	0.534	1.02 (0.97–1.06)	0.425
Female sex	3.48 (1.51–8.04)**	**0.004**	3.49 (1.31–9.33)*	**0.013**
Disease duration, per month	1.02 (1.01–1.04)**	**0.009**	1.03 (1.01–1.05)**	**0.001**
MRSS score	1.01 (0.97–1.06)	0.586	1.05 (1.00–1.10)*	**0.046**
Nutritional markers (Z-scores)				
BMI Z-score	0.92 (0.63–1.35)	0.668	0.74 (0.45–1.21)	0.236
Albumin Z-score	0.85 (0.52–1.38)	0.509	0.75 (0.43–1.30)	0.309
Triglycerides Z-score	1.70 (1.05–2.77)*	**0.032**	1.30 (0.74–2.32)	0.363
Inflammatory markers (Log + Z-scores)				
log(ESR) Z-score	1.27 (0.78–2.04)	0.335	1.82 (1.01–3.27)*	**0.047**
log(SII) Z-score	1.42 (0.89–2.27)	0.137	1.55 (0.92–2.62)	0.102
log(SCI) Z-score	0.78 (0.47–1.29)	0.330	0.81 (0.46–1.42)	0.454
Immune markers				
Anti-Scl-70 positive	3.26 (1.31–8.09)*	**0.011**	2.03 (0.71–5.83)	0.187
Anti-centromere positive	0.19 (0.06–0.68)*	**0.010**	0.26 (0.06–1.12)	0.071
IgA Z-score	0.64 (0.42–0.96)*	**0.031**	0.72 (0.45–1.14)	0.158
Model fit statistics (progressive improvement)				
Model 1 (Baseline only)	AIC = 542.9		Pseudo R^2^ = 0.052	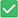
Model 2 (+ Nutritional)	AIC = 496 (A = 46.9)		Pseudo R2 = 0.09 (+ 0.038)	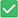
Model 3 (+ Inflammatory)	AIC = 498.1 (A = -2.2)		Pseudo R2 = 0.122 (+ 0.032)	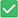
Model 4 (+ Immune/Final)	AIC = 489.1 (A = 9)		Pseudo R2 = 0.156 (+ 0.034)	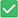

Data are presented as odds ratio (95% confidence interval). Reference group: ILD-/PAH-.

*P < 0.05,

**P < 0.01,

Multinomial logistic regression was performed to identify predictors of the three predominant phenotypes: ILD-/PAH- (reference), ILD + / PAH-, and ILD+/PAH+. The ILD-/PAH + subgroup (*n* = 12) was summarized descriptively (Tables 1–3) but was not included in multivariable modeling due to limited sample size and the resulting risk of unstable estimates (sparse-data bias/separation). Baseline characteristics presented as original values; nutritional and immune markers as Z-scores; inflammatory markers as log-transformed Z-scores to address non-normal distributions. Model comparison shows progressive improvement: AIC decreases and Pseudo R^2^ increases from baseline to final model. Given the small size of the ILD-/PAH + subgroup (*n* = 12), multivariable modeling focused on the three predominant phenotypes; ILD-/PAH + characteristics are shown in Tables 1–3. AIC, Akaike Information Criterion (lower is better); Pseudo R2, McFadden’s R2 (higher is better). Bold values indicate statistically significant differences (*P* < 0.05).

Beyond these shared correlates, domain-specific signals emerged. In the nutritional domain, triglycerides (Z-score) were independently associated with ILD + /PAH- (OR 1.70, 95%CI 1.05–2.77, *p* = 0.032) but not with ILD + /PAH + (*p* = 0.363). In the inflammatory domain, log (ESR) (Z-score) was independently associated with ILD + /PAH + (OR 1.82, 95%CI 1.01–3.27, *p* = 0.047), whereas log(SII) and log(SCI) did not retain independent associations in the final model. Immune markers refined separation between phenotypes. Anti-Scl-70 positivity was independently associated with ILD + /PAH- (OR 3.26, 95%CI 1.31–8.09, *p* = 0.011). ACA positivity showed an inverse association with ILD + /PAH- (OR 0.19, 95%CI 0.06–0.68, *p* = 0.010). IgA (Z-score) was inversely associated with ILD + /PAH- (OR 0.64, 95%CI 0.42–0.96, *p* = 0.031). For ILD+/PAH+, these immune markers showed weaker or non-significant associations in the optimized model (anti-Scl-70 *p* = 0.187; ACA *p* = 0.071; IgA *p* = 0.158). MRSS was associated with ILD+/PAH + (OR 1.05, 95%CI 1.00–1.10, *p* = 0.046) but not with ILD + /PAH-.

Model fit improved with stepwise addition of biomarker domains (AIC decreased from 542.9 in the baseline model to 489.1 in the final model; McFadden’s pseudo R^2^ increased from 0.052 to 0.156), indicating incremental value of integrated biomarkers for phenotype-oriented stratification. After accounting for key clinical factors, ILD + /PAH + is most strongly linked to inflammatory intensity (ESR) and clinical severity (mRSS), whereas ILD + /PAH- is differentiated by a coherent immune–metabolic profile (anti-Scl-70, ACA, IgA, triglycerides).

### Unsupervised clustering: biological validation of clinical phenotypes

**FIGURE 2:**
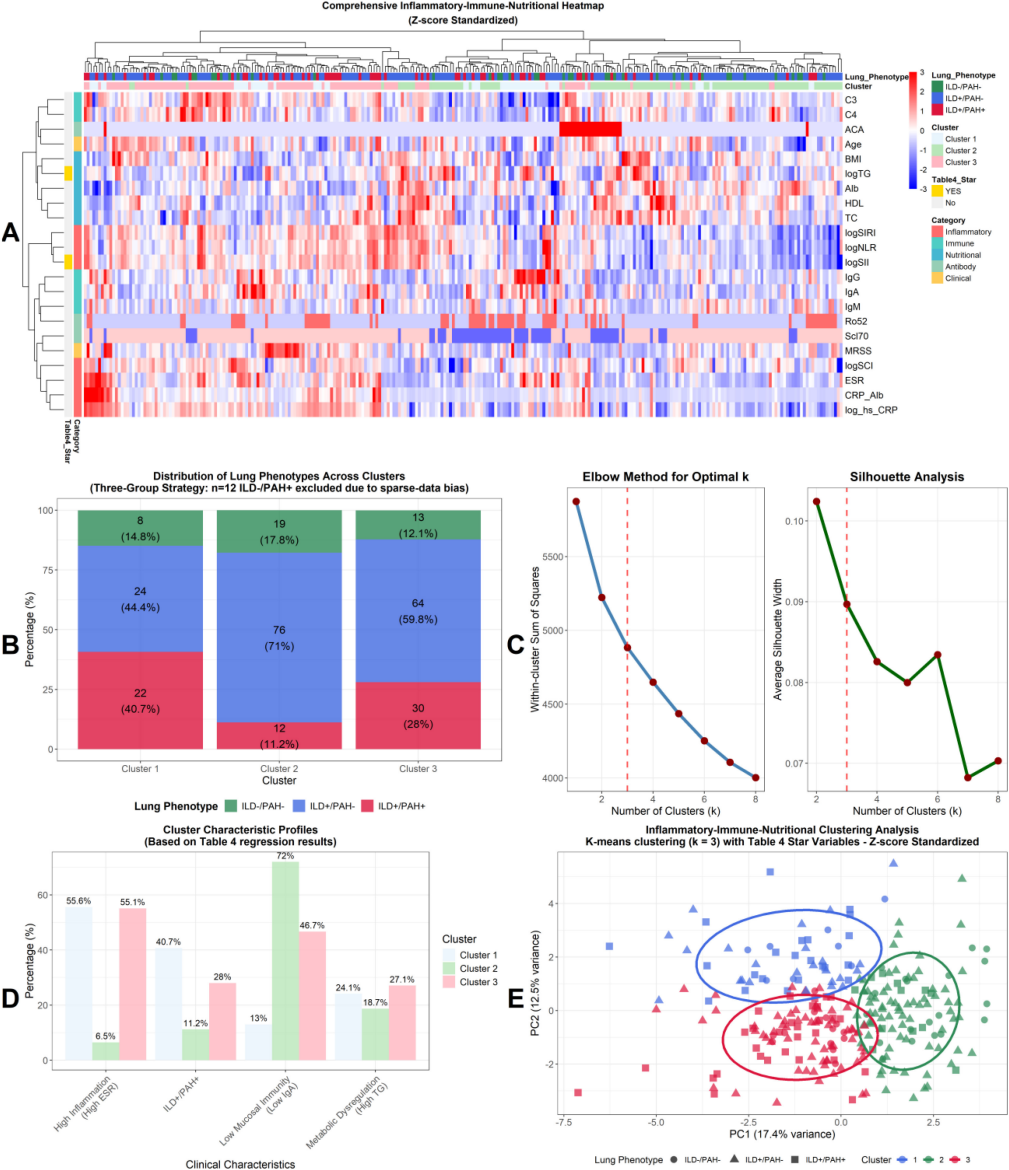
Unsupervised clustering validates the “three-hit” pathogenesis model and reveals distinct phenotypes. **(A)** Hierarchical clustering heatmap of 19 standardized clinical markers. **(B)** Distribution of lung phenotypes across the three identified clusters. **(C)** Optimal number of clusters (*k* = 3) determined by the Elbow method and Silhouette analysis. **(D)** Clinical characteristic profiles revealing distinct biological drivers. **(E)** PCA visualization showing the clear separation of the Mucosal-Inflammatory cluster (Cluster 2) from the PAH-enriched Vascular cluster (Cluster 1).

To test whether integrated biomarker patterns yield data-driven subgroups consistent with the phenotype framework, we performed unsupervised clustering on standardized inflammatory-immune-nutritional markers. The *k* = 3 solution was supported by elbow and silhouette diagnostics (Figure 2C). Hierarchical heatmap visualization demonstrated structured co-variation of biomarker domains across patients (Figure 2A), and PCA showed separation of clusters in low-dimensional space (Figure 2E). Cluster composition differed across phenotypes (Figure 2B). In the three-group clustering strategy (excluding ILD-/PAH + for consistency with the regression approach), clusters showed distinct phenotype enrichments: Cluster 2 contained a high proportion of ILD + /PAH- patients (76, 71.0%), whereas Cluster 1 included a larger fraction of ILD + /PAH + patients (22, 40.7%) alongside ILD-/PAH- (24, 44.4%). Cluster 3 was dominated by ILD + /PAH- (64, 59.8%) with a substantial ILD + /PAH + component (30, 28.0%). Cluster characteristic profiles highlighted three clinically interpretable drivers aligned with the integrated framework: high inflammation (ESR), low mucosal immunity (IgA), and metabolic dysregulation (triglycerides) (Figure 2D).

Clustering stability was high. Across random seeds, clustering consistency was perfect for the *k* = 3 solution (Adjusted Rand Index = 1.00), and bootstrap stability metrics supported robust cluster structure (Supplementary Figure 2). Unsupervised clustering independently recovers stable, interpretable biomarker-defined subgroups aligned with the phenotype framework and highlights three core biological axes—ESR, IgA, and triglycerides—that structure heterogeneity across SSc lung phenotypes.

### Internal validation and sensitivity analyses

Internal validation of the optimized 3-group multinomial model showed stable within-cohort performance. Across validation procedures (bootstrap resampling and 5-fold cross-validation), overall accuracy remained in a relatively narrow range (approximately 0.62–0.65), consistent with a phenotype-oriented stratification objective rather than individual-level prediction. Model performance improved stepwise from baseline clinical variables (Model 1) to the full integrated model (Model 4), consistent with the progressive AIC and pseudo R^2^ improvements reported in Table 4 (Supplementary Figure 1). Internal validation supports the robustness of the phenotype-level associations and the incremental value of integrating biomarker domains, without positioning the model as a standalone clinical prediction tool. Additional validation results are provided in the Supplementary Results.

## Discussion

SSc pulmonary involvement is inherently heterogeneous and has traditionally been studied through fragmented single-endpoint approaches, examining ILD and PAH as separate entities rather than considering their co-occurrence patterns and shared pathobiological mechanisms (1, 19–21). In this cross-sectional study, we reframed SSc pulmonary involvement as a spectrum of four ILD/PAH-based lung phenotypes and demonstrated that these phenotypes correspond to distinct integrated inflammatory-immune-nutritional signatures. Rather than focusing on a single complication or isolated biomarker, we combined routine inflammatory indices, autoantibody profiles, and nutritional-metabolic markers to characterize the systemic milieu associated with each phenotype. The central observation is that ILD + /PAH + represents a high-burden systemic phenotype with consistently elevated inflammatory markers, while ILD + /PAH- is differentiated by a coherent immune–metabolic signature.

A key finding is the convergent evidence for heightened systemic inflammation in ILD + /PAH + . In univariate comparisons, ILD + /PAH + exhibited higher ESR, hs-CRP, CRP/albumin ratio, and SII. In multivariable modeling, log (ESR) remained independently associated with ILD + /PAH + even after adjusting for disease duration and skin severity (mRSS). This suggests that the inflammatory state is an intrinsic biological feature of this phenotype rather than merely a reflection of late-stage disease or fibrotic burden. Clustering analyses similarly highlighted an “inflammation axis” anchored by ESR. Together, these results suggest that concurrent ILD and PAH is not simply the sum of two complications but may represent a systemic inflammatory state in which microvascular injury, immune activation, and fibrotic remodeling interact (22–25). Such an integrated inflammatory milieu plausibly contributes both to progressive interstitial fibrosis and to pulmonary vascular remodeling. Our results also refine established immune associations in SSc. Anti-Scl-70 enrichment in ILD-containing phenotypes observed in Table 3 is consistent with prior literature linking anti-topoisomerase I antibodies with fibrotic lung involvement (26–28). In multivariable analysis, anti-Scl-70 positivity was independently associated with ILD + / PAH-, supporting an ILD-dominant immune signature. Conversely, ACA showed an inverse association with ILD + /PAH- in the adjusted model, aligning with its established relationship to limited cutaneous disease and vascular complications (13–15). While we observed no significant univariate difference in anti-Ro52 prevalence across phenotypes, this antibody is known to be associated with severe ILD (27). Our study was not powered to detect interactions between anti-Scl-70 and anti-Ro52, but future larger cohorts should investigate whether “double-positive” patients represent a distinct high-risk subset. Immunoglobulin differences (IgG/IgA) across phenotypes indicate that humoral immune activation varies across ILD/PAH patterns. Notably, clustering emphasized IgA as a key axis (“mucosal immunity”), supporting its role as an integrative marker distinguishing biological subgroups even when its regression association with ILD + /PAH + was weaker (12, 29, 30). Nutritional and metabolic markers provided complementary differentiation. Triglycerides differed across phenotypes in Table 2 and remained independently associated with ILD + /PAH- in Table 4, while BMI differed across phenotypes in Tables 1, 2 (31, 32). Rather than viewing nutrition-metabolic markers as non-specific, our integrated framework treats them as downstream readouts of chronic inflammation, catabolic state, and systemic metabolic stress. The clustering results further support this view by identifying a metabolic dysregulation axis (high triglycerides) that helps partition heterogeneous patients beyond inflammatory markers alone. Biologically, these clusters suggest distinct pathophysiological pathways: the inflammatory cluster (high ESR) may be driven by systemic cytokine activity (e.g., IL-6) associated with vascular remodeling; the mucosal immunity cluster (low IgA) could reflect gut-lung axis dysregulation; and the metabolic cluster (high triglycerides) points to a metabolic-inflammatory phenotype where lipid dysregulation contributes to endothelial dysfunction.

These findings have clinical implications. Recognizing ILD + /PAH + as a high-burden systemic phenotype characterized by high inflammation supports closer cardiopulmonary surveillance and comprehensive assessment in patients with similar biomarker patterns. Integrated signatures may complement existing screening approaches (e.g., DETECT for PAH) by capturing systemic disease load rather than focusing solely on organ-specific tests (7, 20, 33, 34). Finally, the clustering results raise the possibility that biomarker-defined subgroups could inform phenotype-tailored monitoring and future hypothesis-driven mechanistic studies, although prospective validation is needed.

### Limitations

First, the cross-sectional design precludes causal inference and cannot determine whether signatures precede phenotype development. Second, this was a single-center inpatient cohort, which introduces selection bias toward more severe, inflammatory, and diffuse cutaneous phenotypes. Consequently, the prevalence of ACA (8.6%) was lower than in general SSc populations, and our findings may not fully capture the classic ACA-driven vascular phenotype often managed in outpatient settings. Third, the PAH-only phenotype (ILD-/PAH +) was small, limiting power for multivariable analysis of that subgroup; we addressed this by restricting modeling to the predominant phenotypes while transparently presenting descriptive results for all four groups. Fourth, PAH classification relied mainly on echocardiography, with right heart catheterization available only in a subset, so misclassification is possible (7, 18). Specifically, the ILD + /PAH + group likely comprises a mix of PAH and ILD-PAH, however, identifying the inflammatory signature of this combined high-burden group remains clinically valuable. Finally, treatment exposures were heterogeneous. We did not adjust for immunosuppressive therapy (e.g., glucocorticoids) due to confounding by indication, as sicker patients (ILD + /PAH +) were more likely to receive intensive treatment. Notably, the fact that these patients exhibited the highest inflammatory burden despite likely treatment-induced suppression suggests the underlying biological signal is robust.

Longitudinal studies should evaluate whether integrated inflammatory-immune-nutritional signatures predict transitions between phenotypes, progression, and survival (21, 35). Multi-omics and imaging-linked studies may clarify mechanisms underlying the ESR-IgA-triglyceride axes suggested by our clustering results (12, 25). Prospective work should also test whether biomarker-informed phenotyping can enhance risk stratification and guide management beyond current clinical algorithms (36, 37).

## Conclusion

SSc lung phenotypes defined by ILD and PAH co-occurrence are associated with distinct integrated inflammatory-immune-nutritional signatures. The ILD + /PAH + phenotype emerges as a high-burden systemic inflammatory state, while ILD + /PAH- is differentiated by a coherent immune–metabolic profile. By integrating routine inflammatory indices, autoantibodies, immunoglobulins, and nutritional-metabolic markers, we provide a phenotype-oriented framework that complements traditional organ-based assessments and supports future precision-guided stratification in SSc pulmonary disease.

## Data Availability

The raw data supporting the conclusions of this article will be made available by the author, without undue reservation.
