# Supplementary material for: Integrated inflammatory-immune-nutritional signatures differentiate lung phenotypes in systemic sclerosis
Source: Front Med (Lausanne). 2026 Apr 14;13:1795461. doi: 10.3389/fmed.2026.1795461 (PMC13120917; doi:10.3389/fmed.2026.1795461)
Supplement: Supplementary file 1 [file Data_Sheet_1.docx]

**Supplementary materials**

# Supplementary Results

**Multinomial regression: internal validation and variable importance**

Internal validation of the multinomial logistic regression models is summarized in Supplementary Figure S1. Consistent with the strategy used in the main analysis (Table 4), the ILD-/PAH+ subgroup (n=12) was excluded to mitigate sparse-data bias, focusing the validation on the three predominant phenotypes (n=302). Bootstrap resampling (n=100) yielded stable classification performance, with mean accuracy increasing from 0.615 (Model 1) to 0.645 (Model 4). Five-fold cross-validation produced similar estimates, confirming robustness to sampling variation.

As expected, the stepwise addition of biomarker domains resulted in a progressive improvement in model fit: McFadden’s pseudo R² increased from 0.081 in Model 1 (clinical baseline) to 0.176 in Model 4 (final optimized model). Variable importance analysis, based on the permutation feature importance metric, identified female sex, systemic inflammation (log-transformed SII and ESR), and age as the most influential contributors to phenotype discrimination. Notably, while disease duration was statistically significant in the regression model (Table 4), its lower ranking in the permutation analysis suggests that its predictive information may partially overlap with age and cumulative inflammatory burden in this classification task.


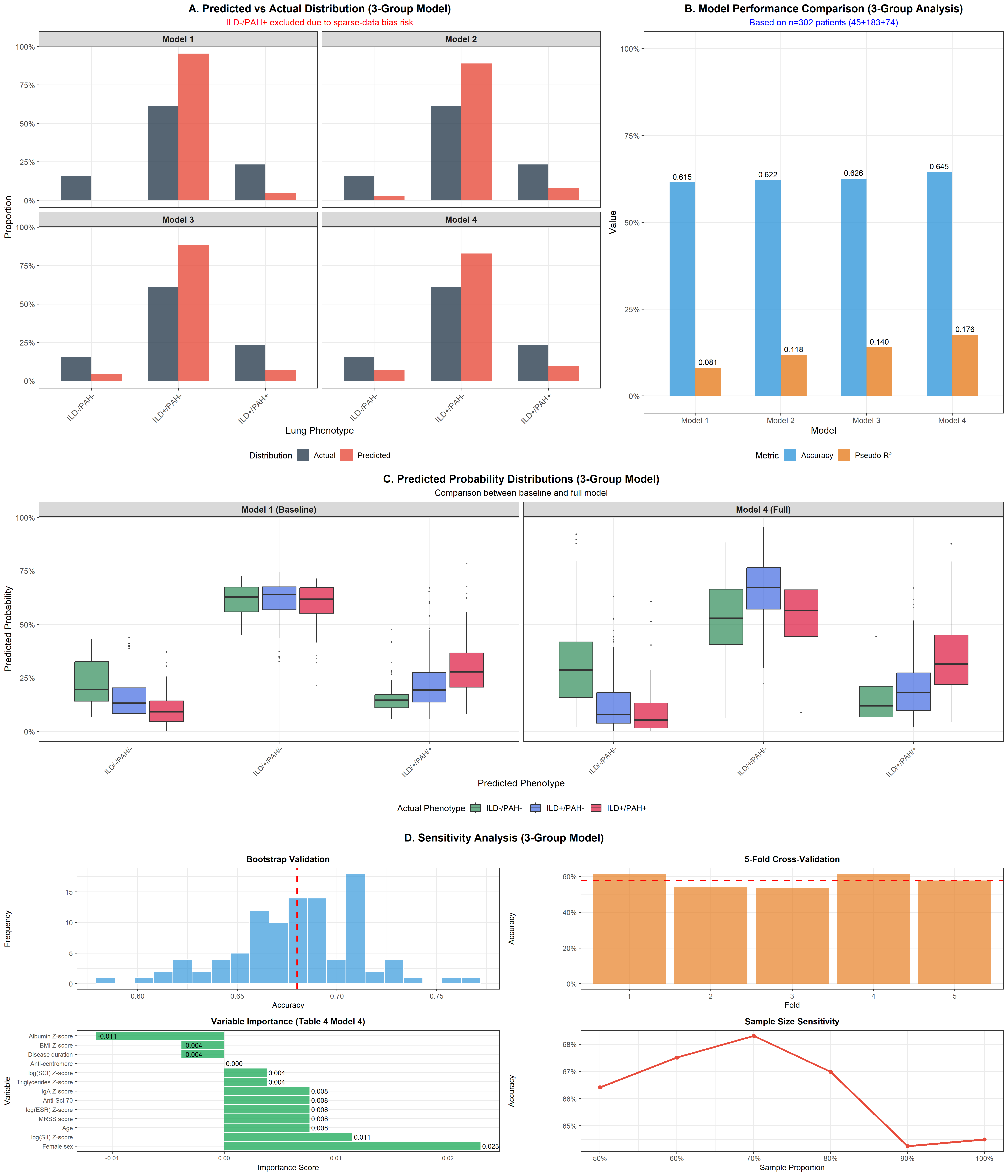


**Supplementary Figure S1. Internal validation of the optimized 3-group multinomial regression**

(A) Predicted vs. actual distribution of lung phenotypes. The analysis was restricted to the three predominant groups (n=302), excluding the ILD-/PAH+ subset consistent with the Table 4 methodology to avoid sparse-data bias. (B) Model performance comparison showing stepwise improvement in Accuracy and McFadden’s Pseudo R² from the baseline clinical model (Model 1) to the final comprehensive model (Model 4). (C) Boxplots of predicted probabilities derived from Model 4. The model assigns higher probabilities to the correct diagnostic categories (matching colors), demonstrating effective discrimination, particularly for the dual-hit ILD+/PAH+ phenotype. (D) Variable importance ranking for the predictors included in the final model (Model 4). Female sex, SII, Age, and ESR emerged as the top contributors to model accuracy.

**Clustering stability and validation**

The robustness of the three-cluster inflammatory-immune-nutritional solution was rigorously evaluated using complementary approaches (Supplementary Figure S2). Gap statistic analysis (Figure S2A), while highlighting the continuous nature of biological data, supported the selection of k=3 as the optimal segmentation strategy consistent with the clinical phenotypes observed in the main analysis. Clustering consistency analysis (Figure S2B) demonstrated exceptional stability, with an adjusted Rand index of 1.00 across 10 random seeds, confirming that the classification is reproducible and independent of initialization.

Furthermore, within-cluster coefficient of variation (CV) analysis (Figure S2C) was performed on the core predictors identified in the regression model. This revealed that while demographic factors (Age, BMI) showed low variability across all groups, the key inflammatory and immune drivers (ESR and IgA) exhibited distinct homogeneity patterns within their respective clusters, validating their role as defining features of the phenotypes. Bootstrap validation (Figure S2D) with 50 resampled datasets yielded narrow distributions for both average silhouette width and within-cluster sum of squares, providing strong data-driven evidence that the identified phenotypes represent genuine biological structures rather than random statistical partitions.


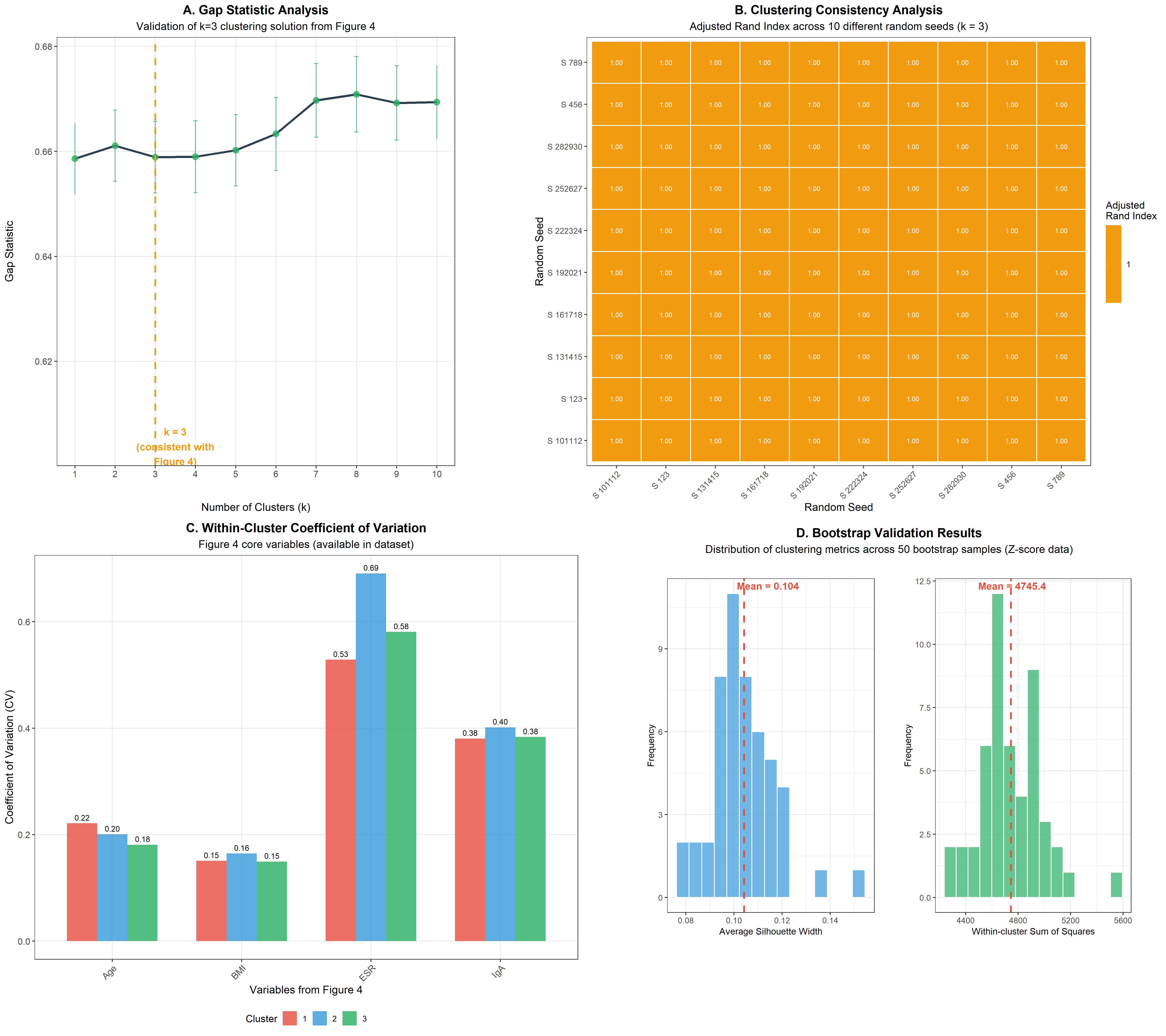


**Supplementary Figure S2. Cluster stability validation**

(A) Gap statistic analysis comparing observed clustering with null reference distributions; the dashed line marks k=3, selected to align with the optimal clinical interpretability and Elbow method results from the main text. (B) Clustering consistency analysis showing perfect stability (Adjusted Rand Index = 1.00) across 10 different random seeds for the k=3 solution. (C) Within-cluster coefficient of variation for core variables used in the Figure 4 model (including Age, BMI, ESR, and IgA). Note that the distinct variability patterns in ESR and IgA support their selection as primary clustering drivers. (D) Bootstrap validation results for 50 resampled datasets (using Z-score standardized data), showing distributions of average silhouette width and within-cluster sum of squares.
